# Lymphoma dissemination is a pathological hallmark for malignant progression of B-cell lymphoma

**DOI:** 10.3389/fimmu.2023.1286411

**Published:** 2023-11-22

**Authors:** Xiaoxi Li, Yong Jiang, Hui Qian

**Affiliations:** Department of Laboratory Medicine, School of Medicine, Jiangsu University, Zhenjiang, Jiangsu, China

**Keywords:** extranodal lymphoma, lymphoma dissemination, lymphocyte homing, benign hyperplasia, lymphoma homing, lymphoma non-lymphoid organ colonization, lymphoma extranodal colonization, reshaping cellular plasticity

## Abstract

Extranodal lymphoma occurs in one-third of lymphoma patients and is a key indicator of the international prognostic index, associated with unfavorable outcomes. Due to the lack of ideal models, the causes and characteristics of extranodal lymphoma are greatly underexplored. Recently, we observed a high incidence of extranodal lymphoma in two types of mouse models with tropism for the brain and kidneys. These findings prompt us to rethink the pathological progression of lymphoma colonization in lymph nodes and non-lymphoid organs. Nodal lymphoma, primary extranodal lymphoma and secondary extranodal lymphoma should be biologically and clinically distinctive scenarios. Based on the observations in mouse models with extranodal lymphoma, we propose that lymphoma dissemination can be seen as lymphoma losing the ability to home to lymph nodes. The pathological process of nodal lymphoma should be referred to as lymphoma homing to distinguish it from benign hyperplasia. Lymphoma dissemination, defined as a pathological process that lymphoma can occur in almost any part of the body, is a key pathological hallmark for malignant progression of B-cell lymphoma. Reshaping cellular plasticity is a promising strategy to allow transformed cells to homing back to lymph nodes and re-sensitize tumor cells to treatment. From this perspective, we provide new insights into the pathological progression of lymphoma dissemination and its inspiration on therapeutic interventions. We believe that establishing extranodal lymphoma mouse models, identifying molecular mechanism governing lymphoma dissemination, and developing therapies to prevent lymphoma dissemination will become emerging topics for fighting relapsed and refractory lymphoma.

## Introduction

Lymphoma is a highly heterogeneous lymphoid disease. Diffused large B-cell lymphoma (DLBCL) accounts for one-third of B-cell lymphoma and is the most extensively studied. About 60% of DLBCL patients can be cured with R-CHOP (rituximab, cyclophosphamide, doxorubicin, vincristine and prednisone) immunochemotherapy, while the others cannot benefit (1). Relapesed/refractory DLBCL usually occurs together with lymphoma dissemination. If lymphoma dissemination is observed in patients at the early stage of diagnosis, such as central nervous system (CNS) involvement, high-dose chemotherapy regimens will be administrated (2). In this case, high-dose chemotherapy, such as Methotrexate (MTX) and Cytarabine (Ara-C), will be used to treat, which is believed to pass through the Blood-Brain Barrier (BBB) and reach lethal dosage at the site of lymphoma. Unfortunately, so far, there has been no significant improvement in the survival of patients with extranodal lymphoma in clinical trials. One possible explanation is that the damage caused by lymphoma dissemination to affected organs is lethal and irreversible, so inhibiting lymphoma growth is not enough to improve patient health. Moreover, the adverse effects of chemotherapy on immune system may promote malignant progression of lymphoma.

Transcriptome and genomic studies have greatly promoted the molecular classification of DLBCL, which help to understand the pathogenesis mechanism of lymphoma and development of targeted drugs. Based on gene expression profiles, DLBLC can be divided into the activated B-cell-like (ABC) subtype and the germinal center B-cell-like (GCB) subtype, which is known as Cell of Origin (COO) classification (3). Patients with ABC subtype are characterized by activation of B-cell Receptor (BCR) and nuclear factor-kappa B (NF-κB) signaling pathways and have poorer prognosis than patients with GCB subtype. Extranodal lymphoma mainly occurs in the ABC subtype. Based on genetic variations, DLBCL can be divided into 7 subtypes, which is known as LymphGen classification (4), and extranodal lymphoma mainly occurs in MCD subtype, which is characterized by MYD88^L265P^ and/or CD79B mutations. Similar to ABC subtype, activation of BCR and NF-κB signaling pathways is the main feature of the MCD subtype. Therefore, targeted therapies blocking the key kinases in BCR and NF-κB signaling pathways, such as BTK and PI3K, are considered promising strategies to improve the prognosis of relapsed/refractory DLBCL (5). It is worth noting that activation of BCR and NF-κB signaling pathways is indeed necessary to maintain cell survival and proliferation, but may not be the main cause of lymphoma dissemination. The genetic and non-genetic mechanism for regulating lymphoma dissemination is still largely unknown. Exploring the biological mechanism of lymphoma dissemination and establishing nodal and extranodal lymphoma models will provide important insights for the prevention and treatment of extranodal lymphoma.

## Rethinking the Eμ-Myc transgenic mouse model

Since the genetic background of inbred mouse strains is stable and well-defined, genetically engineered mouse (GEM) models are considered as ideal research tools to explore pathological mechanism of lymphoma. Eμ-Myc transgenic mouse is one of the most widely studied GEM models for B-cell lymphoma. However, this model has some drawbacks, such as high heterogeneity and the cell of origin at early B-cell stage, which challenge its reliability as a human lymphoma model.

Although Eμ-Myc transgenic mouse is initially designed to resemble Myc translocation observed in human Burkitt Lymphoma (BL) (6), lymphomas arising in Eμ-Myc mice exhibit significant inter-tumor heterogeneity and both BL-like and DLBCL-like gene expression patterns are observed (7–9). The two-hit theory (10) suggests that the initiation of Eμ-Myc lymphoma requires deletion or loss of function (LOF) mutation of additional tumor suppressor genes (TSGs). Early studies reported that some TSGs, such as Arf and p53, were randomly inactivated in Eμ-Myc lymphoma (11–14) and knocking out these TSGs could significantly accelerate lymphoma initiation (15, 16). In addition, a recent study revealed that Bcor was randomly inactivated in Eμ-Myc lymphoma and was a Myc co-operative tumor suppressor gene (17). Therefore, the secondary genetic aberrations that occur randomly contribute to the heterogeneity of Eμ-Myc lymphoma.

Eμ-Myc mice have a high incidence of spontaneous lymphoma, but lymphomas arising in Eμ-Myc mice originate from the pre-B to mature B-cell stages, predominantly being the pre-B/immature B-cell stage (18–20). Given that human B-cell lymphomas predominantly originate from the mature B-cell stage (18, 21), Eμ-Myc lymphomas are considered as abnormal proliferation of the early B-cells and Eμ-Myc models are not recognized as the faithful model resembling human BL or DLBCL. It is worth noting that the frequency of mature B-cell lymphomas is significantly increased in a few Eμ-Myc derived mouse models, and one of them is the Eμ-Myc;Utx^KO^ model we previously reported (22). The Eμ-Myc;Utx^KO^ model can develop mature B-cell lymphoma and extranodal lymphoma, which are similar to human aggressive mature B-cell lymphoma. Many studies have shown that UTX deletion can promote malignant progression in various tumor mouse models (23–25), confirming that the loss of UTX can be seen as a critical secondary mutation in the two-hit theory of cancer. Genomic studies have shown that a large number of epigenetic regulatory genes undergo high-frequency mutations in lymphoma and other tumors (26), indicating that non-genetic mechanisms are key mechanisms promoting malignant transformation of tumors. Given that the biological function of UTX is mainly demethylation at H3K27, a key epigenetic mechanism on gene transcription regulation, the malignant transformation of tumor is likely not due to the loss of UTX itself, but rather due to the widespread transcriptional abnormalities caused by the loss of UTX function. The epigenetic changes caused by UTX deletion and resulting transcriptional changes may regulate genes involving lymphoma dissemination, thereby can be seen as key events leading to lymphoma dissemination. In summary, although Eμ-Myc mice predominantly develop pre-B/immature B-cell lymphoma, mature B-cell lymphoma can indeed occur randomly in Eμ-Myc derived mouse models. Therefore, we believe that Eμ-Myc derived mouse models can serve as faithful models for human B-cell lymphoma.

Due to homogeneity and tractability, cell-line derived allografts (CellDAs) models (27) rather than GEM models are generated for therapeutic intervention studies. Eμ-Myc;Cdkn2a^-/-^ cell line is a widely used murine lymphoma cell line to establish the CellDAs model and Eμ-Myc;Cdkn2a^-/-^ lymphomas usually form palpable masses at multiple lymph nodes (28, 29). We recently identified a cell line, named MA, which originates from Eμ-Myc;Cdkn2a^-/-^ cell line. M refers to Myc gene and A refers to Arf gene (30). Different from Eμ-Myc;Cdkn2a^-/-^ lymphomas, MA lymphomas arising in recipient mice usually colonize in kidney, and at the same time, lymph nodes are often unaffected. In contrast to nodal lymphoma, renal tropism of MA lymphoma greatly affects the survival of recipient mice and the feasibility of further treatment. Although we do not know the reason for renal tropism of MA lymphoma, we believe that the MA cell line had undergone transcriptome evolution, supporting the idea that non-genetic mechanisms may be the key reason for promoting malignant transformation. Furthermore, the MA cell-line transplant model indicates that lymphoma cells themselves are sufficient to lead to lymphoma dissemination behavior, suggesting that the MA cell line can be regarded as a transformed form of Eμ-Myc;Cdkn2a^-/-^ cell line. Moreover, the generation of the MA primary renal lymphoma model provides a tractable tool for resembling aggressive B-cell lymphoma and investigating pathological progression of extranodal lymphoma.

## Lymphoma dissemination is caused by the loss of homing ability of lymphoma cells

First of all, we need to clarify several basic concepts used in this article. Lymphoma dissemination refers to the phenomenon that lymphoma can occur in almost any part of the body, including septate and non-septate lymphoid tissues, non-lymphoid tissues and organs. Nodal lymphoma refers to lymphoma that occurs in lymph nodes and originates from lymph nodes. Specifically, it is a cancer that occurs in lymph nodes, often causing lymph node enlargement and potentially disseminating to other parts of the body over time. Extranodal lymphoma refers to lymphoma that appears in extranodal locations, including primary extranodal lymphoma and secondary extranodal lymphoma. Lymphoma arising in non-septate mucosa-associated lymphoid tissues, such as mucosa-associated lymphoid tissue lymphoma (MALT), a subtype of marginal zone lymphoma (MZL), is beyond the scope of this article. Aggressive lymphoma can be either nodal lymphoma or extranodal lymphoma. In most cases, lymphoma dissemination indicates aggressive lymphoma, but in a few cases, extranodal lymphoma also can be indolent lymphoma. Lymphoma dissemination is one of the prognostic indicators for lymphoma, but it is not necessary for malignant progression of lymphoma. Whether lymphoma has undergone malignant progression requires a comprehensive judgment based on multiple features of lymphoma, and lymphoma dissemination is one of the most prominent features of malignant progression of lymphoma. It should be noted that the malignant progression of lymphoma we mentioned here refers to the transition of lymphoma from a slow-growing, slow-progressing state to a rapidly growing, rapidly progressing state, which is different from the concept of malignant transformation in solid tumors. Malignant transformation in solid tumors refers to the transformation of benign proliferating cells into malignant tumors with invasiveness.

**Figure 1:**
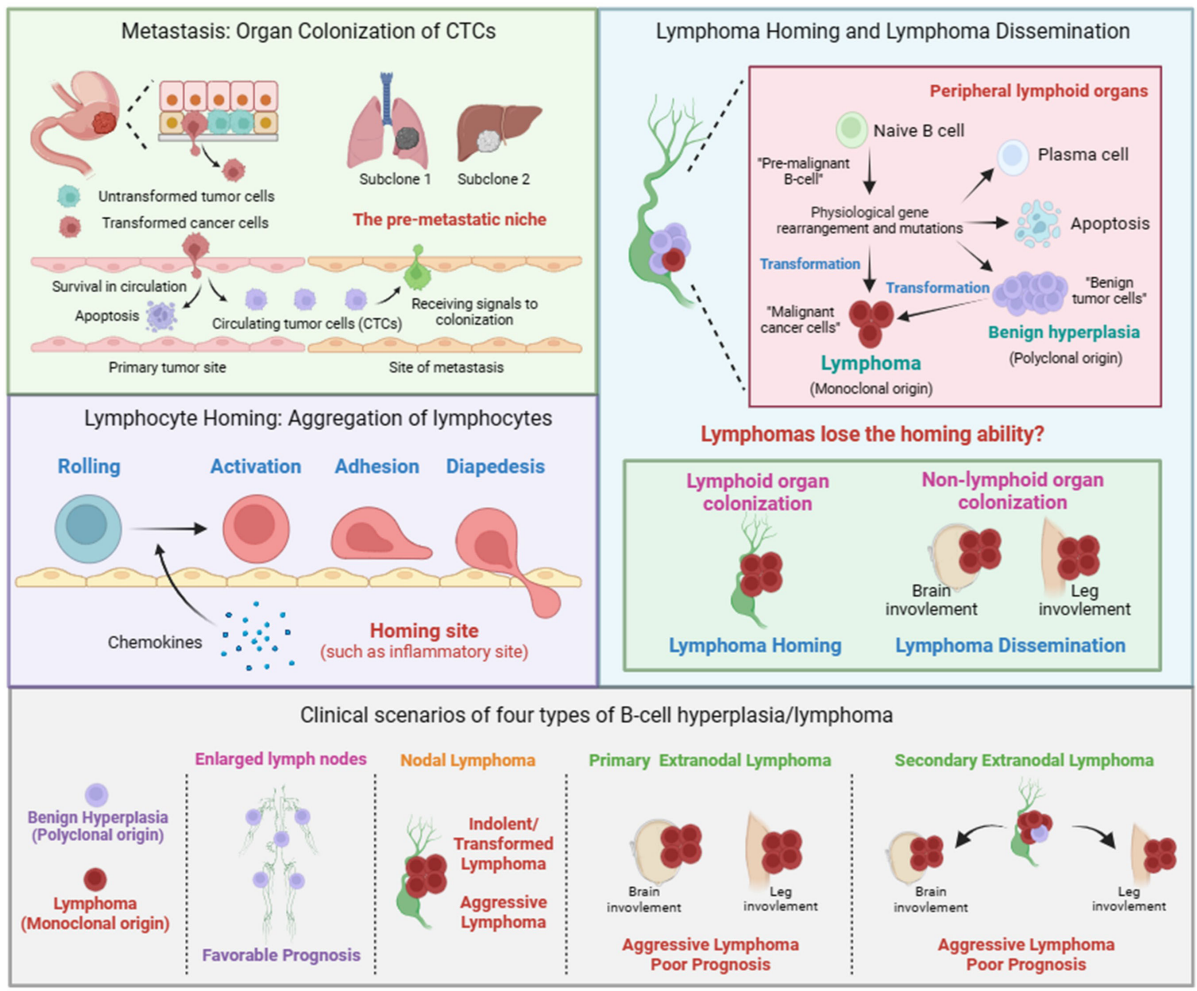
Metastasis of solid tumor, lymphocyte homing, lymphoma homing and dissemination. Metastasis of solid tumor includes the formation of circulating tumor cells (CTCs) and the colonization of CTCs. The colonization of CTCs is facilitated by mulitiple factors, including tumor cells and the pre-metastastic niche in disseminated organs. Lymphocyte homing includes rolling, activation, adhesion, and diapedesis. The term lymphoma homing is used to describe the pathological process of nodal lymphoma. The term lymphoma dissemination is used to describe the pathological process of extranodal lymphoma. Given the polyclonal nature, we believe that the pathological process of benign B-cell hyperplasia is similar to that of lymphocyte homing. Clinical scenarios of four types of B-cell hyperplasia/lymphoma. Nodal lymphoma includes indolent, transformed, and aggressive lymphoma. Primary or secondary extranodal lymphoma is usually classified as aggressive lymphoma with poor prognosis.

To illustrate the biological significance of lymphoma dissemination, we firstly review the current knowledge of pathological progression of solid tumors. Metastasis usually means that the tumor is difficult to eradicate through surgery, making it a hallmark of malignant transformation of solid tumors (31). Metastasis is a multistep process and generally includes three steps (**Figure 1**). The first step is the acquisition of invasiveness, which refers to the ability of some tumor cells to spread from where they started, enter the bloodstream, and circulate throughout the body. The second step is the survival in circulation, which refers to circulating tumor cells (CTCs) undergoing selection and adaptation in the bloodstream. The third step is organ colonization, which refers to the colonization of CTCs to distant organs. The colonization of CTCs is facilitated by multiple factors, including tumor cells and the pre-metastastic niche in disseminated organs. CTCs can guide the formation of pre-metastatic niche in disseminated organs by emitting signals, such as exosomes. It should be note that the pathological progression of solid tumors and lymphomas is not completely the same. Tumor transformation refers to the process of benign hyperplasia turning into malignant tumor/cancer. For solid tumors, invasion and metastasis are the fundamental differences between benign and malignant tumors. For lymphoma, the difference between benign hyperplasia and lymphoma mainly lies in the clonality of neoplasm. Lymphomas originate from monoclonal proliferation, and in addition to excessive proliferation or apoptosis defects, they also have additional malignant features, such as lymphoma dissemination proposed in this perspective.

Unlike solid tumors, lymphoma cells have already been in circulation. Therefore, lymphoma cells do not need to invade the primary site to enter the blood circulation, as is the case with solid tumors. The process of lymphoma cells developing into lymphoma in extranodal tissues or organs is more similar to the process of malignant solid tumor cells colonizing at distant sites. Considering the homing behavior of lymphocytes, the current view is that lymphoma dissemination is a physiological behavior similar to the homing of lymphocytes, rather than malignant progression (32). In certain physiological or pathological situations, such as inflammation, lymphocytes will aggregate to specific locations, which is called homing. Homing is a multistep process including rolling, activation, adhesion, and diapedesis (**Figure 1**). The signals emitted by the homing site, such as chemokines, can promote the activation of lymphocytes and guide their colonization, which is similar to the signal released by the pre-metastatic niche guiding tumor cell colonization. Therefore, lymphoma dissemination is considered to be similar to lymphocyte homing. The self-activation signal and external signals from the site of colonization lead to changes in the transcription programs within lymphoma cells, affecting the expression of cell adhesion-related genes such as integrins, which ultimately enables lymphoma cells to acquire the ability to colonize specific tissues or organs.

In this perspective, we propose that, in addition to homing to specific sites guided by the released external signals, lymphocytes themselves also possess homing ability, mainly homing to lymphoid tissues. Similarly, lymphoma cells should also have homing ability and stay in lymphoid tissues. Therefore, lymphoma dissemination should be a result of the loss of homing ability of lymphoma cells. In addition, given that lymphoma is also a special solid tumor, there should be many similarities between the lymphoma dissemination and the colonization of CTCs. Together, we adopted the terms “homing and colonization” and gave them new connotations to describe the process of lymphoma dissemination as following (**Figure 1**).

We propose that the term nodal/extranodal colonization is used to describe the pathological process of nodal/extranodal lymphoma, and the term lymphoma homing/dissemination is used to describe the pathological mechanism of nodal/extranodal lymphoma. Whether lymphoma colonizing in lymph nodes or colonizing in extranodal tissues or organs depends on whether lymphoma loses its homing ability during its progression. Lymphoma that does not lose its homing ability is colonized in lymph nodes, which is known as nodal lymphoma. Lymphoma that loses its homing ability will colonize in various parts of the body, which is known as extranodal lymphoma. Given the polyclonal nature, we believe that the pathological process of benign B-cell hyperplasia is similar to that of lymphocyte homing.

Here, we clarify that the concept of lymphoma dissemination is similar to solid tumor metastasis. Furthermore, we also clarify the concepts of indolent lymphoma, transformed lymphoma, and aggressive lymphoma, and their correlation with lymphoma dissemination. In clinical diagnosis, lymphoma can be broadly classified into indolent lymphoma and aggressive lymphoma based on the growth rate and disease progression of lymphoma. Follicular lymphoma (FL) is a type of indolent lymphoma. DLBCL, BL are two types of aggressive lymphoma. Indolent lymphoma may also accumulate additional genetic aberrations, leading to rapid growth and acquisition of additional malignant characteristics of lymphoma. This situation is known as transformed lymphoma. Therefore, the concepts of indolent, transformed, and aggressive lymphoma are mainly used to describe and evaluate the growth rate and progression of lymphoma. However, the degree of malignancy of lymphoma should not be limited to the growth rate of lymphoma. The site of dissemination is also a key indicator for determining the prognosis of lymphoma. According to the locations of lymphoma, it can be divided into nodal lymphoma and extranodal lymphoma. Lymphoma dissemination is a description of the colonization ability of aggressive lymphoma. Lossing the homing ability is one of the possible outcomes of lymphoma progression and an important reason of poor prognosis, but it is not necessary for aggressive lymphoma. Therefore, we conclude that lymphoma dissemination often indicates a poor prognosis, and a poor prognosis often accompanies lymphoma extranodal colonization.

Given that the process of solid tumor colonization is believed to be guided by signals secreted by the pre-metastatic microenvironment, we do not exclude that extranodal colonization of lymphoma may also be regulated by external signals. However, lymphoma itself that lost the homing ability should be the major reason for lymphoma dissemination. We hope that these concepts can help understand and describe the process of the lymphoma dissemination.

## The evolution of lymphoma dissemination: lessons from mouse models

We mainly observed four types of growth sites of lymphoma in Eμ-Myc GEM models and cell-line transplant models (**Supplemental Image 1**). The Eμ-Myc model and Eμ-Myc; Cdkn2a^-/-^ cell transplant mice usually develop enlarged lymph nodes at cervical, axillary, inguinal. No obvious blood vessels were found on the surface of lymphomas, and the health status of mice is not affected by enlarged lymph nodes. At the same time, no infiltration of other important organs and tissues were found. Although these lymphomas had proliferation advantages/apoptosis defects, they had the homing ability and can only spread within the lymph nodes/lymphoid system, similar to the untransformed tumor. Together, these lymphomas within multiple lymph nodes involved should be benign B-cell hyperplasia, but lymphoma. It should be noted that the original Eμ-Myc mice with hybrid backgrounds usually develop extranodal lymphoma (6, 19), largely different from what we observed in inbred C57BL/6 mice, indicating that the genetic background of mice used in the experiments is one of the factors affecting pathological progression of lymphoma. Therefore, we propose that the C57BL/6 Eμ-Myc mice with multiple enlarged lymph nodes should be regarded as a model of pre-transformed/benign B-cell hyperplasia disease, but not a faithful aggressive lymphoma model.

In Eμ-Myc;Utx^KO^ model and MA transplant model, three types of growth sites of lymphoma occur randomly. The first is the appearance of only one nodal lymphoma with obvious blood vessels, usually an axillary lymph node was involved. The second is the appearance of only extranodal lymphoma. The third is the concurrence of single nodal lymphoma and extranodal lymphoma, but it is difficult to distinguish whether the extranodal lymphoma is primary or secondary. The health status of the mouse is poor in all three situations. These three situations are considered to be aggressive lymphoma, mainly due to the fact that the lymphoma has not only gained proliferative advantages but also is not confined to the lymph nodes/lymphoid system, lost the homing ability, and has entered the bloodstream, are finally disseminated to extranodal tissues/organs through hematogenous dissemination. Human aggressive B-cell lymphoma in the clinic, such as BL, DLBCL, are more similar to the aggressive lymphoma observed in Eμ-Myc;Utx^KO^ model and MA transplant model.

According to cell clonality, homing ability, and disease progression, lymphoma can be classified into four categories (**Figure 1**). 1), Polyclonal benign B-cell hyperplasia typically leads to multiple lymph nodes enlargement. 2), lymphomas had undergone dissemination/transformation and presented in one lymph node, known as primary nodal lymphoma, including indolent, transformed or aggressive lymphoma. 3), lymphomas had undergone dissemination/transformation and presented in extranodal locations, known as primary extranodal lymphoma/aggressive lymphoma. 4), lymphomas had undergone dissemination/transformation in one lymph node and then disseminated to extranodal locations, known as secondary extranodal lymphoma/aggressive lymphoma.

Lymphoma dissemination in different anatomic sites should involve different pathological progressions. We propose that there should be two models of lymphoma extranodal colonization. One model is that lymphoma cells lose their homing ability and randomly colonize in extranodal locations. The other is that lymphoma cells acquire the ability to colonize in specific organs during the malignant progression. These two models should not be mutually exclusive and are applicable to different organs respectively. Although the losing homing ability is not necessary for malignant progression of B-cell lymphoma, in most cases, extranodal lymphoma is more common in aggressive B-cell lymphoma and should be an indicator of malignant progression of B-cell lymphoma.

There are three main evolutionary models for extranodal lymphomas in human lymphoma: direct evolution, branching evolution, and convergent evolution (4). But these phylogenetical models are mainly inferred based on the proportion of mutations in clinical samples of lymphoma. There is a lack of experimental validation to determine whether these mutations are driver genes that can significantly promote tumor progression and clonal evolution.

Unlike clinical samples, mouse models have a clear genetic background, making it easier to analyze the impact of genetic factors on tumor progression, especially malignant progression. Considering the clonal expansion of lymphoma, we speculate that the evolutionary relationship between nodal lymphoma and extranodal lymphoma should be direct evolution, fitting the two-hit theory. Taken together, overexpression of Myc drives the pre-malignant B-cell progenitor initially to benign B-cell hyperplasia, and a transformed clone, for example driven by loss of UTX, then make the benign B-cell hyperplasia into nodal lymphoma and/or further spread to extranodal sites to form extranodal lymphoma. The direct evolution model also fits our theory that extranodal lymphoma is the further deterioration of nodal lymphoma, which loses its homing ability during the malignant progression.

A thought-provoking question is what the loss of homing ability in lymphoma cells means for the pathological progression of lymphoma and the clonal evolution. Here, we hypothesize two models for the loss of homing ability. One model is that lymphoma cells lose their homing ability passively. During the rapid growth of lymphoma, genetic mutations in lymphoma cells continue to accumulate, which may lead to abnormal lymphoid-specific transcription programs and affect molecules that maintain lymphocyte functions such as homing, resulting in the loss of homing ability in lymphoma cells. Another model is that lymphoma cells actively lose their homing ability. During the rapid growth of lymphoma, lymphoma cells require more nutrients for protein synthesis and cell proliferation. Compared to the lymphatic system, the blood system has more oxygen and nutrients, and lymphoma cells have a stronger proliferative advantage in the blood. Therefore, lymphoma cells that lose their homing ability are more likely to be selected and adapted during tumor clonal evolution. We believe that these two models are not mutually exclusive and may coexist. The loss of homing ability is not only a manifestation of the abnormal regulatory network in lymphoma, but also facilitates the adaptation of lymphoma to maintain a homeostasis for rapid proliferation. Therefore, the loss of homing ability is an advantage acquired by lymphoma cells during the evolutionary process.

## Potential prevention and intervention strategies for lymphoma dissemination

Lymphoma dissemination is associated with poor prognosis. On the one hand, extranodal lymphoma can impair involved organs. On the other hand, drugs cannot reach killing dosage in involved organs. Moreover, extranodal lymphoma itself may develop intrinsic chemoresistance. Therefore, understanding the difference of nodal lymphoma and extranodal lymphoma is of great significance in fighting relapsed/refractory lymphoma.

**Figure 2:**
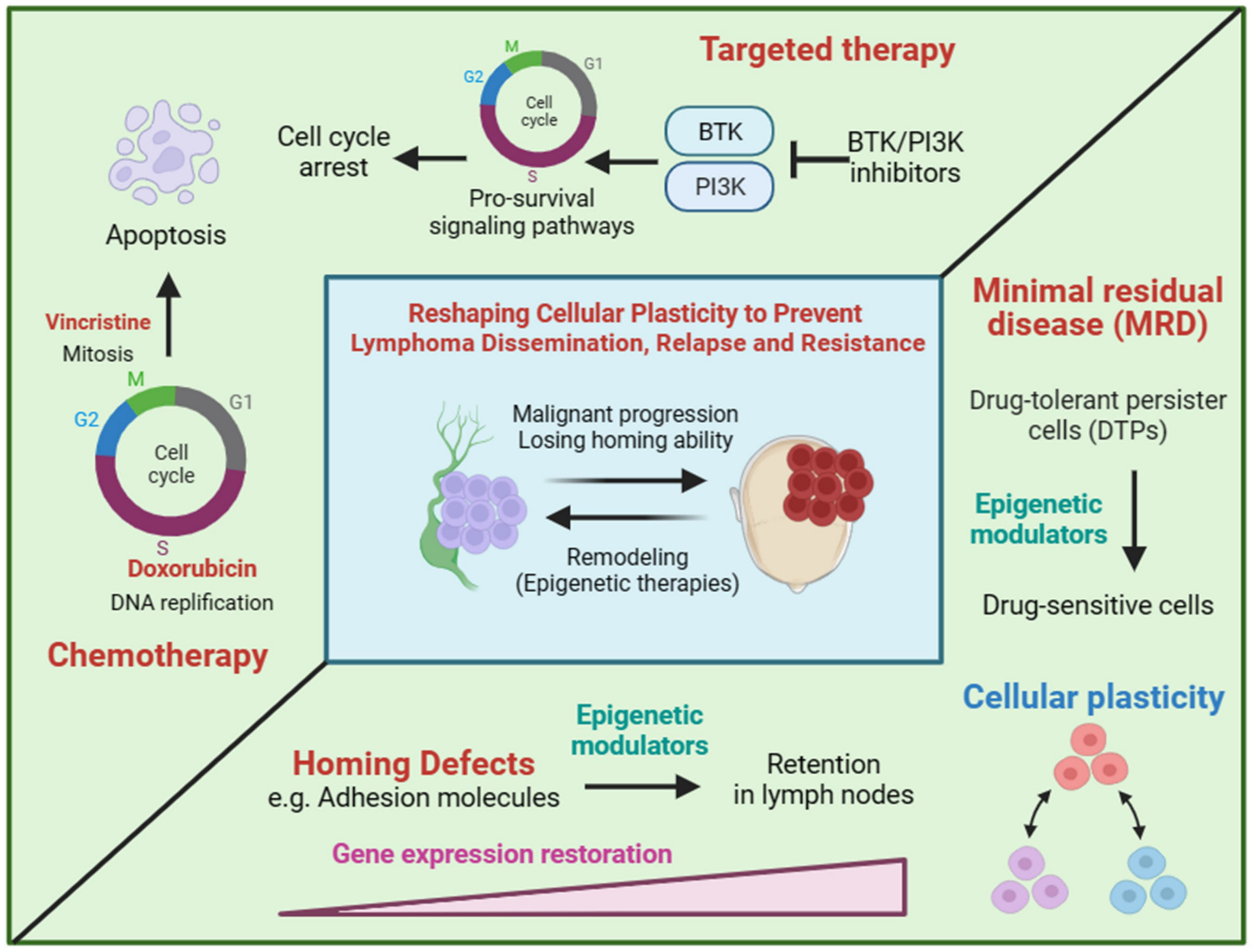
The hypothesis of reshaping cellular plasticity to govern lymphoma dissemination. Chemotherapy and targeted therapy are designed to kill proliferating cells by inducing cell cycle arrest and apoptosis. Drug-tolerant persister cells (DTPs) represent non/low proliferating cells in the presence of drugs. DTPs is often pre-existing in tumor cell population and subsequently selected by treatment, which is clinically defined as minimal residual disease (MRD). Cell plastisity refers to the ability of cells to transform from one phenotype to another in response to external stimuli. For example, rapid proliferating cells and non/low proliferating cells are both pre-existing in a tumor population and some of rapid proliferating cells can convert to non/low proliferating cells in the present of treatment. Similarly, we hypothesis that extranodal/nodal colonization of lymphoma is a reflection of cell plastisity and can be regulated by transcriptional programming of genes involving homing to lymph nodes. Therapies that reshaping cellular plasticity, such as epigenetic modulators, are expected to re-sensitize DTPs by converting DTPs to rapid proliferating cells as well as preventing lymphoma dissemination by reprogramming homing related genes.

Chemotherapy and targeted therapy are main treatments for lymphoma. Chemotherapy drugs induce apoptosis of rapidly proliferating cells through blocking DNA synthesis or mitosis in the cell cycle. Targeted drugs suppress cell proliferation by inhibiting the pro-survival and pro-proliferation signaling pathways (**Figure 2**). Hence, non/low proliferating cells will be less responsive to chemotherapy and targeted therapy. Tumor heterogeneity is the main reason of chemoresistance. Given that lymphoma originates from a monoclone, the heterogeneity of lymphoma may be mainly caused by non-genetic mechanism. Cellular plastisity refers to the ability of cells to transform from one phenotype to another in response to external stimuli and is controlled by non-genetic mechanism (33–36). Drug-tolerant persister cells (DTPs) are a type of non/low proliferating cells with intrinsic chemoresistance in tumor cell population and subsequently selected by treatment, which is clinically defined as minimal residual disease (MRD) (**Figure 2**). The generation of DTPs is also a reflection of cellular plasticity. The loss of homing ability, the silencing of homing related genes regulated by non-genetic mechanism, also can be regard as a reflection of cellular plasticity. We hypothesis that extranodal lymphoma have a CSCs-like or DTPs-like tumor cell population, therefore extranodal lymphoma itself can develop intrinsic chemoresistance.

Given tumor cellular plasticity, the homing ability and cell proliferation of tumor cells may be mainly regulated by non-genetic mechanism. Therefore, inducing or blocking gene expression may be potential strategies for prevention and intervention of extranodal lymphoma (**Figure 2**). Epigenetic modulators and inhibitors of master transcriptional factors are two treatment categories of gene expression regulation. DNA methyltransferase inhibitors (DNMTi) and histone deacetylase inhibitors (HDACi) are two kinds of epigenetic modulators, both of which are believed to activating gene expression by transiting chromatin from a closed state into an open state. Therefore, epigenetic modulators may restore the expression of homing related genes and educate lymphoma cells to homing back to lymph nodes (**Figure 2**). However, due to the global gene regulation, tumor-promoting genes involving homing-inhibition genes may also be activated by epigenetic modulators. Hence, efficacy of epigenetic modulators to prevent lymphoma dissemination need to be evaluated in preclinical animal models. Master transcriptional factors manipulating a few genes usually have better specificity, which makes them more promising drug targets. However, transcriptional factors governing lymphoma dissemination are largely unknown. Similarly, the reversal of cellular plasticity may also re-sensitize lymphoma cells to chemotherapy and targeted therapy by licensing cells to re-enter cell cycle.

In summary, we propose that lymphoma dissemination and cell proliferation status are two manifestations of cellular plasticity of lymphoma cells. The reversal of cellular plasticity by epigenetic modulators can be a potential prevention and intervention strategy for lymphoma dissemination. Master transcriptional factors governing lymphoma dissemination are also potential drug targets and should be identified in the future.

## Discussion

Lymphoma dissemination is an important feature of B-cell lymphoma, but it has not been extensively studied as a pathological process of lymphoma. Lymphoma dissemination presents high randomness in both human patients and mouse models, which makes it look less like a controllable process. Although genomic studies indicate extranodal lymphoma is mainly enriched in the MCD subtype/ABC subtype, this only suggests that these genetic variations and transcriptional patterns are correlated with extranodal lymphoma, there is currently no direct causal evidence of genetic variations causing extranodal lymphoma. The molecular characteristics of the MCD subtype and the ABC subtype, such as deletion of CDKN2A, BCL2 overexpression, activation of BCR and NF-κB signaling pathways, may be the cause and result of lymphoma losing homing ability. Considering the high frequency of mutations in epigenetic regulatory genes in lymphoma and various cancers, such as UTX, EZH2, MLL2, we speculate that abnormal epigenetic regulation, a non-genetic mechanism, may be a key mechanism that promotes malignant progressionof lymphoma and the loss of homing ability. We believe that identifying key molecules and mechanisms that directly affect the biological process of lymphoma dissemination of lymphoma, such as EFNB1 identified in the UTX study, is of great significance for understanding the pathological mechanisms of lymphoma and preventing lymphoma dissemination to improve patient prognosis.

Extranodal lymphoma mainly occurs in the MCD subtype, and most of them belong to the ABC origin. Compared to GCB, ABC is a post-GC stage. We speculate that B cells acquire the homing ability during the GC stage, that is when naive B cells enter lymphoid organs from the blood, and that the loss of homing ability is involved in the transcriptome changes during the post-GC stage. Many malignant B-cell lymphomas of GC origin, such as BL and FL, occur in lymph nodes. And many malignant B-cell lymphomas of post-GC origin, such as DLBCL-ABC, MZL, disseminate to extranodal organs/tissues, such as CNS, gastrointestinal tract, urogenital system, mucosa, skin, etc. In line with this, in Eµ-Myc;Utx^KO^ mouse models with high incidence of extranodal lymphoma, most of the lymphoma also originate from mature B-cell stage. Therefore, we believe that lymphoma mouse models with high incidence of extranodal lymphoma, such as Eµ-Myc;Utx^KO^ model and MA transplant model, should be recognized as the faithful model resembling human aggressive B cell lymphoma. It should be noted that cancer is a disease caused by the accumulation of random mutations. In experimental models, the accumulated mutations vary between mice/lymphomas, leading to differences in their transcriptional profiles and cell of origins. A study (37) described that individual B-cell lymphomas shared only a quarter of all differentially expressed genes. Therefore, it is difficult to define or predict the cell of origin of lymphomas arising in spontaneous lymphoma mouse models, accounting for the heterogeneity of spontaneous lymphoma mouse models. Leukemia originating from mature B cells, such as chronic lymphocytic leukemia (CLL), may also be found in the Eμ-Myc model.

For a long time, lymphoma dissemination has been considered a physiological behavior rather than a reflection of lymphoma progression, resulting in a lack of attention to the phenotype of lymphoma dissemination in existing mouse models. Many mature B cell-specific knockout GEMMs have been developed, which are regarded as faithful human lymphoma models (18), but there is little description of extranodal lymphoma in these models. It should be noted that some studies have reported histologically detectable infiltration of lymphomas in the liver and lung, which is largely far from primary extranodal lymphoma observed in the clinic. One study (38) reports that TBL1XR1 mutations can drive extranodal lymphoma with a memory B-cell origin in VavP-Bcl2;CD19-Cre;Tbl1xr1^KO/KO^ mice. Another study (39) reports that extranodal lymphoma occurs in a CNP-Cre-driven sleeping beauty (SB) mutagenesis mouse model. Interestingly, similar to Eμ, CNP is also expressed in early B-cell progenitors. From our experience in lymphoma models, although rare and random, the occurrence of lymphoma dissemination seriously threatens the survival and therapeutic effectiveness of lymphoma mouse models. Therefore, we suggest that the evaluation criteria for lymphoma mouse models should shift from GEM models with genetic backgrounds similar to human DLBCL to mouse models with behavioral characteristics of aggressive B-cell lymphoma, such as lymphoma dissemination. In addition, lymphoma mouse models based on non-genetic mechanisms, gene expression regulation rather than gene deletion or mutation, should also be an important direction for the development of lymphoma mouse models in the future.

Preclinical mouse models are mainly designed for evaluating the effect of candidate drugs or therapies on inhibition of lymphoma growth, rather than their effect on lymphoma dissemination. This is actually not consistent with clinical scenarios. In clinical trials, participants are usually unable to benefit from standard treatment, partly due to the destructive effect of extranodal lymphoma on colonized organs. Therefore, preventing and intervening in lymphoma dissemination, rather than simply inhibiting lymphoma growth, is more likely to improve patient prognosis. As Pals ST et al. mentioned, “However, retention and expansion per se can hardly explain the strikingly different dissemination patterns of the distinct lymphoma subtypes. Moreover, to our knowledge, there are currently no studies available that address the role of retention regulation in lymphoma dissemination, preventing a meaningful discussion of this aspect” (32). The lack of models for lymphoma dissemination may be the main reason for limiting relevant studies. Given the clinical significance of extranodal colonization in lymphoma progression, extranodal lymphoma models should be an important direction for the development of lymphoma mouse models.

In this perspective, according to the observations from Eμ-Myc derived models and the inspirations from metastasis of solid tumors, we rethink the pathological progression of lymphoma dissemination. We propose that lymphoma non-lymphoid organ colonization is due to lymphoma losing the ability to homing to lymph nodes. Lymphoma dissemination, defined as a pathological process of lymphoma non-lymphoid organ colonization, is a key pathological hallmark for malignant progressionof B-cell lymphoma. Reshaping cellular plasticity may allow tumor cells to homing back to lymph nodes and re-sensitize tumor cells to treatment, making it a promising strategy for fighting relapsed/refractory lymphoma and improving the survival of patients with extranodal lymphoma.

## Data availability statement

The original contributions presented in the study are included in the article/**Supplementary Material**. Further inquiries can be directed to the corresponding authors.

## Author contributions

XL: Conceptualization, Funding acquisition, Supervision, Visualization, Writing – original draft, Writing – review & editing. YJ: Writing – review & editing. HQ: Writing – review & editing.

## Supplementary material

Supplemental Image 1 Lymphoma dissemination in Eμ-Myc derived GEM models and CellDAs models. The Eμ-Myc transgenic mice and recipient mice of the Eμ-Myc; Cdkn2a^-/-^ cell line usually develop multiple enlarged lymph nodes at mandibular, axillary, inguinal regions. The Eμ-Myc; Utx^KO^ mice and recipient mice of the MA cell line usually develop extranodal lymphoma at brain, thymus, kidney, and/or one nodal lymphoma with most cases being axillary.
