# Supplementary figures and images for: Lymphoma dissemination is a pathological hallmark for malignant progression of B-cell lymphoma
Source: Front Immunol. 2023 Nov 22;14:1286411. doi: 10.3389/fimmu.2023.1286411 (PMC10703179; doi:10.3389/fimmu.2023.1286411)

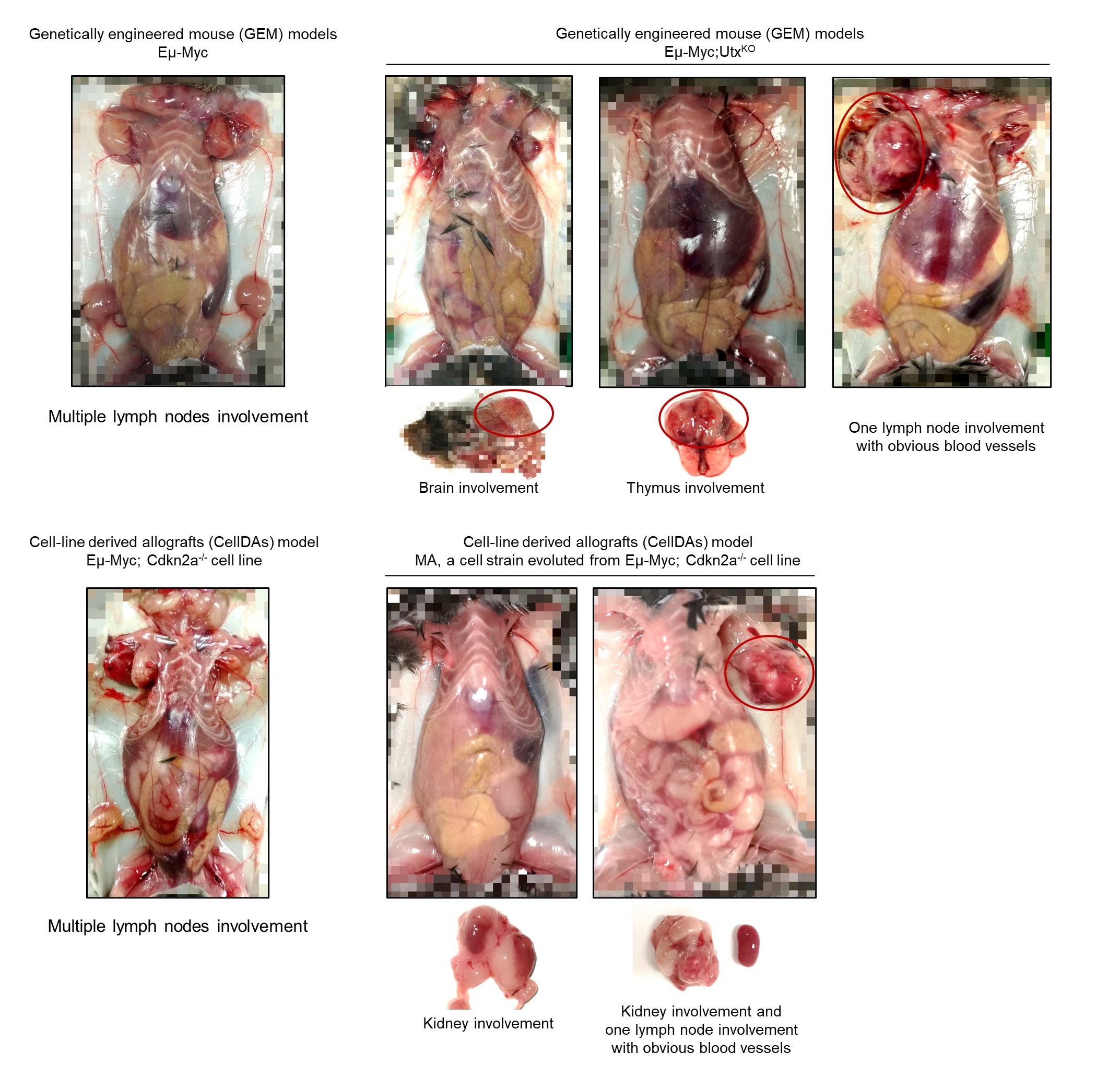

Supplement: Supplemental Image 1 — Lymphoma dissemination in Eμ-Myc derived GEM models and CellDAs models. The Eμ-Myc transgenic mice and recipient mice of the Eμ-Myc; Cdkn2a-/- cell line usually develop multiple enlarged lymph nodes at mandibular, axillary, inguinal regions. The Eμ-Myc; UtxKO mice and recipient mice of the MA cell line usually develop extranodal lymphoma at brain, thymus, kidney, and/or one nodal lymphoma with most cases being axillary. [file Image_1.jpeg]
